# Genetic Mismatches Between Nuclei and Mitochondria Make Yeast Hybrids Sterile

**DOI:** 10.1371/journal.pbio.1000433

**Published:** 2010-07-20

**Authors:** Robin Meadows

**Affiliations:** Freelance Science Writer, Fairfield, California, United States of America

**Figure pbio-1000433-g001:**
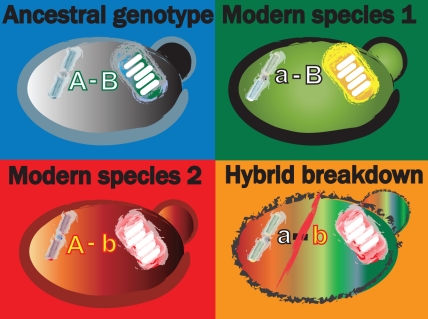
Is the fast-evolving mitochondrial genome a driving force of speciation? Image: S-L Chang.

[Fig pbio-1000433-g001]When one species mates with another, the resulting hybrids typically die or fail to reproduce. Hybrids can form between similar species from microorganisms to mammals, with the mule being a classic example. Sterile offspring of horses and donkeys, mules are prized for being more agile than the former, less obstinate than the latter, and smarter than both. But researchers prize hybrids for an additional reason: these reproductive dead ends could help explain how new species begin to form. A critical mechanism on the path to speciation is known as genetic incompatibility, in which genes from diverging species no longer interact properly. These improper interactions prevent these species from producing fertile offspring, leading to reproductive isolation.

Understanding the basis of genetic incompatibility is key to identifying the driving forces of speciation. In a new study in this issue of *PLoS Biology*, Jun-Yi Leu and colleagues address the role of incompatibility between genomes in the nucleus and mitochondria in generating reproductive isolation in yeast.

Mitochondria have a limited genome that produces a mere eight of the 1,000-some proteins this organelle needs to function, leaving the overwhelming majority to be encoded in the nucleus. Nuclear-mitochondrial genetic incompatibility occurs broadly amongst organisms including plants, insects, amphibians, and primates, yet this intracellular conflict is not well understood at the molecular level.

Although Baker's yeast (*Saccharomyces cerevisiae*) mates readily with several close relatives, nearly all the gametes (spores) from these hybrids die. The researchers reported in 2008 that hybrids of *S. cerevisiae* and *Saccharomyces bayanus* exhibited a nuclear-mitochondrial mismatch (known as cytonuclear incompatibility): an *S. bayanus* nuclear gene (*AEP2*) blocked translation of an *S. cerevisiae* mitochondrial mRNA.

In this study, Leu and colleagues set out to determine whether cytonuclear incompatibility is a common cause of reproductive isolation in yeast by investigating hybrids of *S. cerevisiae* (Sc) crossed with either *S. bayanus* (Sb) or *Saccharomyces paradoxus* (Sp). One parent in each cross was a mutant that effectively lacks mitochondria, thus allowing the researchers to track which species contributed these organelles to the hybrids. Mitochondria are key players in respiration, the process whereby cells produce energy, and growth assays revealed that most of the hybrid spores were respiration-deficient, indicating cytonuclear incompatibility. The worst cytonuclear mismatch was between Sc nuclei and Sb mitochondria, where about two-thirds of the spores failed to respire.

Next, the researchers identified the genes causing this mismatch by screening for those that rescued respiration in the hybrid spores. In Sc nucleus-Sb mitochondria hybrids, respiration was restored by two Sb nuclear genes required for mitochondrial function, showing that the corresponding Sc nuclear genes were incompatible with Sb mitochondria. The genes were *AIM22*, which encodes a ligase required for mitochondrial protein lipoylation, and *MRS1*, which encodes a protein (Mrs1) required to remove an intron from the mitochondrial *COX1* gene.

Sc is the most recently evolved species, and two lines of evidence suggest that its Mrs1 protein has lost the ability to splice the mitochondrial *COX1* introns in the other two yeast species. First, in mutants of the other two species that lack this protein, transcription of the Sc *MRS1* gene and transport of the resulting protein into mitochondria were normal. Second, comparison of *COX1* introns from a range of yeast species showed that Sc lacks an ancient intron found in the rest.

To pinpoint why the protein from the Sc nuclear gene is incompatible with this ancient mitochondrial intron, the researchers engineered Mrs1 proteins that had bits from both Sc and one of the other yeast species. Engineered proteins that were compatible with the ancient intron had three mutations in a 63-amino acid stretch; a computer model of the protein's structure revealed that these mutations are in the RNA-binding part of the protein.

To trace the evolution of cytonuclear mismatches in yeast hybrids, the researchers engineered the nuclear genes (*MRS1*, *AIM22*, and *AEP2*) that are incompatible with mitochondrial genes into the three closely related yeast species. The pattern of cytonuclear mismatches indicated that they occurred at different times in different lineages over the course of yeast evolution.

The repeated evolution of nuclear-mitochondrial genetic mismatches suggests that this mechanism may play a major role in the reproductive isolation of yeast species. Mitochondrial DNA accumulates mutations far faster than nuclear DNA, partly because cells have far fewer mitochondrial genome molecules. This makes cytonuclear incompatibility a likely general mechanism of reproductive isolation, and it will be interesting to see whether this holds broadly across species, as well as between different populations of the same species.

**Chou J-Y, Hung Y-S, Lin K-H, Lee H-Y, Leu J-Y (2010) Multiple Molecular Mechanisms Cause Reproductive Isolation between Three Yeast Species. doi:10.1371/journal.pbio.1000432**

